# Echocardiographic screening in pediatric asymptomatic or paucisymptomatic coronavirus disease 2019 outpatients: is it a useful test or an excess of zeal?

**DOI:** 10.2459/JCM.0000000000001549

**Published:** 2023-08-08

**Authors:** Paolo Alberto Gasparini, Elisa Lodi, Eleonora Rodighiero, Jonathan Rosero Morales, Giuseppe Fantini, Maria Grazia Modena

**Affiliations:** a University of Modena and Reggio Emilia, CHIMOMO Dept c/o Policlinico di Modena; b P.A.S.C.I.A. Center (Heart Failure Care Program, Childhood Heart Diseases and Those at Risk), University of Modena and Reggio Emilia, AOU Polyclinic of Modena, Modena, Italy

**Keywords:** coronavirus disease 2019, echocardiographic screening, pediatric

## Abstract

**Introduction:**

Data regarding echocardiographic findings during follow-up of asymptomatic or pauci-symptomatic coronavirus disease 2019 (COVID-19) are scarce in pediatric patients. The aim of the present study is to assess post-COVID-19 sequelae through echocardiography in children who have experienced mild SARS-CoV-2.

**Methods:**

This single-center, retrospective, observational study enrolled a cohort of 133 pediatric outpatients, born between 2005 and 2022, with a history of asymptomatic or paucisymptomatic SARS-CoV-2 infection, who underwent transthoracic echocardiographic (TTE) evaluation at an outpatient pediatric clinic in Northern Italy.

**Results:**

The percentage of the pediatric activity of the clinic which was focused on post-COVID evaluation was not negligible, representing almost 10% of the ∼1500 pediatric patients examined from 1 January 2021 to 31 August 2022. According to ACEP classification, children enrolled in this study had previously experienced in 72.9% (97) asymptomatic COVID-19 and nearly 27% (36) a mild illness. Clinical and instrumental examinations did not show any relevant abnormality in the functional [left ventricular ejection fraction (LVEF), tricuspid annular plane systolic excursion (TAPSE), pulmonary artery systolic pressure (PASP)] or structural [interventricular septum diameter (IVSd), left ventricular internal diameter (LViD, end-diastolic volume (EDV), left atrium volume (LAV)] parameters examined related to SARS-CoV-2 infection in the total of 133 children.

**Conclusion:**

According to our results, children who experienced an asymptomatic or mild SARS-CoV-2 infection should not be systematically investigated with second-level techniques, such as TTE, in the absence of clinical suspicion or other risk conditions such as congenital heart diseases, comorbidities or risk factors.

## Introduction

Coronavirus disease 2019 (COVID-19) is an infectious disease caused by SARS-CoV-2, and it quickly became a pandemic,^1^ challenging healthcare systems^2–4^ and affecting the lives of billions of people worldwide. From 31 December 2019, when a cluster of pneumonia with unknown cause was first reported in Wuhan City (https://www.who.int/csr/don/05-january-2020-pneumonia-of-unkown-cause-china/en/),^5^ to 31 December 2022, in Italy, almost 25 million cases had been confirmed and ∼180 000 patients have died (https://coronavirus.jhu.edu/region/italy).

Children infected with SARS-CoV-2 were less expected to develop severe COVID-19, with a death rate about 25 times lower than adults’^6^ and deaths from COVID-19 in children about 0.17 per 100 000 population^7,8^; nevertheless, they were still at risk of developing severe illness and complications from COVID-19 (https://www.cdc.gov/coronavirus/2019-ncov/hcp/pediatric-hcp.html). Cardiac involvement in a COVID-19 pediatric patient was usually present when multisystem inflammatory syndrome (MIS-C) developed.^9^ The main symptoms were fever, gastrointestinal disorders, left ventricular systolic dysfunction and finally, shock; additionally, pleural, pericardial and ascitic effusions, along with elevated inflammatory markers had also been identified.^10^ Among MIS-C patients, the heart was often severely impaired. Children might experience an acute cardiac decompensation caused by severe inflammatory state after SARS-CoV-2 infection; for example, in the study of Belhadjer *et al.*^11^ left ventricular ejection fraction under 30% was identified in one-third of the hospitalized pediatric patients enrolled and B-type natriuretic peptide (BNP) and inflammation markers were elevated and suggestive of cytokine storm. To treat children with MIS-C critical care support, vasopressors and mechanical ventilation were often required.^12^ Similar findings have been collected in other studies; for example, in the one conducted by Whittaker *et al.*^13^ almost half of the children affected by MIS-C developed shock and required inotropic support and about 15% developed coronary artery dilatation or aneurysm. However, in recent years, growing evidence about COVID-19 and its possible cardiopulmonary complications has raised concerns about potential subclinical heart damage even in asymptomatic patients.

In addition to the acute phase of COVID-19, long-COVID is a heterogeneous disorder that could affect patients previously infected with SARS-CoV-2, although only a small percentage of children displayed post-COVID-19 sequalae.^14^ Symptoms of pediatric long-COVID were the same as described in adult patients: fatigue, dyspnea, palpitations, myalgia, chest and joint pain and headache; it is notable that children recovering from paucisymptomatic COVID-19 might develop these persisting symptoms.^15,16^

Data regarding pediatric echocardiographic findings during asymptomatic or paucisymptomatic COVID-19 follow-up are scarce. Physicians may have concerns about the most appropriate diagnostic workup, being debated between the risk of potential subclinical heart damage even in asymptomatic patients and the need for appropriate use of the limited resources of the health system.

The aim of the present study is to assess echocardiographic post-COVID sequelae in children who experienced a mild infection.

## Methods

This single-center, retrospective, observational study enrolled a cohort of 133 pediatric outpatients with previous asymptomatic or mild SARS-CoV-2 infection, who underwent transthoracic echocardiographic (TTE) evaluation at an outpatient pediatric clinic in Northern Italy from 1 January 2021 to 31 August 2022 to detect COVID-19 sequelae.

The inclusion criteria were: age under 16 years; previous laboratory-confirmed SARS-CoV-2 infection by antigenic detection in the upper respiratory tract swab test; previous asymptomatic or mild SARS-CoV-2 infection according to ACEP (https://www.acep.org/corona/covid-19-field-guide/diagnosis/diagnosis-when-there-is-no-testing/) (American College of Emergency Physician) classification, where asymptomatic stands for someone who is ‘positive for SARS-CoV-2 using a test but no symptoms that are consistent with COVID-19’ and mild illness refers to a patient who presents ‘signs and symptoms of COVID-19 but no shortness of breath, dyspnea, or abnormal chest imaging’.

Exclusion criteria were: known previous cardiovascular comorbidity; previous COVID-19-related MIS-C; suboptimal TTE image quality.

TTE was performed using pediatric probe S8.3. Echocardiographic images were obtained, with standard transducer positions, in the parasternal long-axis, short-axis, apical four chambers and subxiphoid long-axis and short-axis.

Continuous variables were expressed as mean ± 1 SD and categorical data as percentages.

The present study was conducted according to the principles of the Declaration of Helsinki and was approved by the ‘Ethical Committee of Area Vasta Emilia Nord (AVEN)’.

All patients were informed about their participation in the study and provided oral informed consent for the anonymous publication of scientific data. Written consent to participate was not needed because of the nature of the study: anonymous, retrospective and observational.

## Results

A cohort of 133 pediatric outpatients with previous asymptomatic or mild SARS-CoV-2 infection, who underwent TTE evaluation from 1 January 2021 to 31 August 2022 to detect COVID-19 sequelae were enrolled.

The median age of the cohort was 9.01 ± 2.4 years (year of birth between 2005 and 2022), with a male-to-female ratio of almost 2 : 1 (84 boys, 49 girls).

According to ACEP classification, of the 133 children enrolled in this study, 97 (72.9%) experienced asymptomatic COVID-19 and 36 (27.1%) a mild form of COVID-19.

Eight patients (6%) in the cohort showed possible long-COVID signs and symptoms:

(1) Thoracic pain: 4
(2) Palpitation at rest: 1
(3) Dyspnea at rest: 1
(4) Premature supraventricular beats: 1
(5) Persistent fatigue: 1

The percentage of the pediatric activity of the clinic, which was focused on post-COVID-19 evaluation, was not negligible, representing almost 10% of the ∼1500 pediatric patients examined from 1 January 2021 to 31 August 2022. Not only primis pediatricians but also general practitioners and exercise and sports physicians sent pediatric patients of this cohort to our outpatient cardiological clinic to assess possible COVID-19 cardiac sequelae.

Clinical and instrumental examination did not show any hemodynamically relevant abnormality both in the functional [left ventricular ejection fraction (LVEF), tricuspid annular plane systolic excursion (TAPSE), pulmonary artery systolic pressure (PASP)] and structural [interventricular septum diameter (IVSd), left ventricular internal diameter (LViD), end-diastolic volume (EDV), left atrium volume (LAV)] parameters examined related to SARS-CoV-2 infection in the total of 133 children of the cohort, as shown in Table 1. From a functional point of view, mean LVEF was 61.7 ± 3.4%, mean TAPSE 26.2 ± 1.5 mm, and mean PASP 22.5 ± 1.2 mmHg. From a structural point of view, mean IVSd was 6.2 ± 1.6 mm, mean LViD 34.4 ± 2.4 mm, mean EDV 64.1 ± 3.2 ml and mean LAV 25.4 ± 1.6 ml.

**Table 1:** Echocardiographic parameters

Echocardiographic parameters	Mean value (±SD)	Median value
IVSd (2D)	6,2 ± 1.6 mm	6 mm
LViD (2D)	34.4 ± 2.4 mm	35 mm
EDV (2D)	64.1 ± 3.2 ml	65.5 ml
LAV (2D)	25.4 ± 1.6 ml	24 ml
TAPSE	26.2 ± 1.5 mm	26.5 mm
PASP	22.5 ± 1.2 mmHg	22.5 mmHg
EF	61.7 ± 3.4%	61.5%

EDV, end-diastolic volume; EF, ejection fraction; IVSd, interventricular septum diameter; LAV, left atrium volume; LViD, left ventricular internal diameter; PASP, pulmonary artery systolic pressure; TAPSE, tricuspid annular plane systolic excursion.

**Fig. 1:**
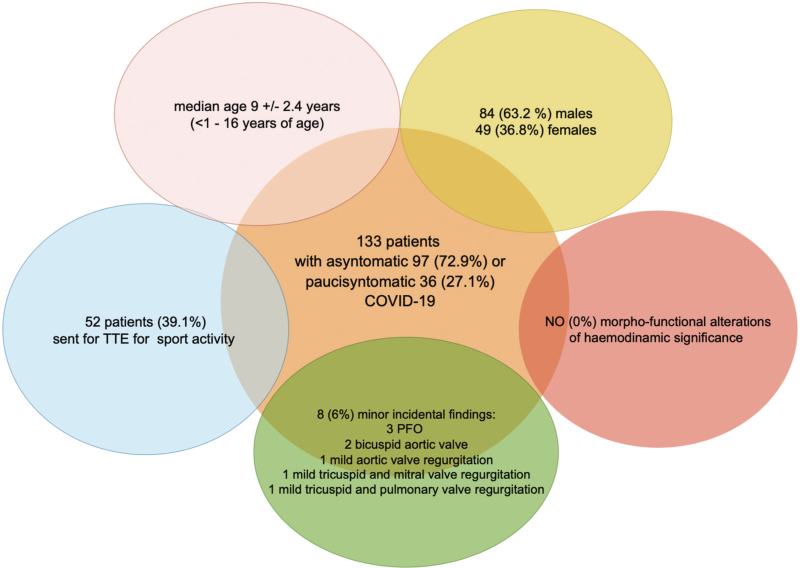
Indications for transthoracic echocardiography, and data of patients and findings of transthoracic echocardiography.

As shown in Fig. 1, incidental findings were described in 6% (eight) of the cohort and were represented by: three patent foramen ovale (PFO) (a physiological condition, which is described in almost 25% of the population^17^), two bicuspid aortic valve (1 type 1 and 1 type 2), one mild aortic valve regurgitation, one mild tricuspid and mitral valve regurgitation, and one mild tricuspid and pulmonary valve regurgitation. None of these had any hemodynamic significance. Even speculating a possible correlation between COVID-19 and the aforementioned valvular alterations, which has yet to be demonstrated, only 2.2% of our cohort was affected, without clinical repercussions, so that a follow-up was not recommended.

## Discussion

In recent years, growing evidence about COVID-19 and its possible cardiopulmonary complications has raised concerns about potential subclinical heart damage even in asymptomatic patients. It is known that heart involvement in COVID-19 pediatric patients is usually rare and minimal. Data about cardiac involvement in patients with asymptomatic or mild COVID-19 are scarce, especially among subgroups such as young pediatric patients and infants. If studies describing MIS-C-related problems are excluded, those that included previously asymptomatic or paucisymptomatic COVID-19 patients enrolled mostly athletes, who underwent cardiological assessment for ‘return to play’ (RTP), and pediatric patients or young adults presenting a median age older than our cohort.^18,19^ As sport activity may trigger adverse events in individuals with cardiovascular abnormalities, many countries worldwide promptly provided recommendations for a safe RTP and sports activity for athletes with previous COVID-19 disease. Italy was among the first nations to deal with the problem of protecting athletes’ health after COVID-19. After an initial version was released in April 2020, in which TTE was recommended to all athletes who underwent SARS-CoV-2 infection, on December 2020, the Italian Sports Medicine Federation (FMSI) updated the recommendations for the RTP of nonprofessional athletes. In the latest version, TTE is not routinely indicated in athletes with a history of asymptomatic or paucisymptomatic (mild illness) COVID-19, as execution of TTE is recommended based on the stratification of the clinical and instrumental risk of the athletes.^20–22^ A position paper from the Dutch Sports Cardiology,^23^ Section of the Netherlands Society of Cardiology, regarding athletes aged more than 16 years states that for those with systemic features, ECG testing should be considered before confirming their readiness to RTP, while preparticipation (PPS) screening of athletes after an asymptomatic or mild SARS-CoV-2 infection is not suggested if a critical evaluation of signs and symptoms is negative and shows a complete recovery, as the chance of cardiac sequelae is probably insignificant. The Dutch position paper also asserts that a PPS among asymptomatic patients might be considered only for those with preexistent cardiovascular disorder and elite athletes.

When symptoms are present and persist, a conservative attitude may be recommended; for example, an evaluation with a sports and exercise medicine physician was advised even after mild symptomatic infection, performing TTE and lung function testing where cardiopulmonary symptoms were present.^24^

In our cohort, 52 (39.1%) of the patients were sent for TTE for a safe return to physical activity, and the remaining 60% to assess possible COVID-19 subclinical cardiac sequelae.

No statistically significant difference was found among patients without symptoms vs. patients with symptoms in the echocardiographic parameters as no morphofunctional abnormalities in the standard parameters analyzed (IVSd, LViD, EDV, LAV, LVEF, TAPSE, PASP) were detected (Table 1).

According to our study, consistently with the limited international literature published nowadays on individuals of the age considered, TTE should be reserved for pediatric patients with clinical signs, several risk factors or severe comorbidities. In fact, also among children who suffered from myocarditis MIS-C related, ergo with an exceptionally more severe cardiac involvement than those among our cohort, when performing cardiac magnetic resonance structural sequelae were rarely described, and there was usually a full cardiac functionality recovery.^25,26^

However, some studies suggested that even in patients with normal LVEF and TAPSE subclinical echocardiographic signs of myocardial dysfunction after COVID-19 might be detected; that is, studies using 2D speckle tracking showed that left ventricle global longitudinal strain (LV-GLS) was significantly lower^27^ and the prevalence of regional peak systolic strain below −16% in at least two segments was higher in patients with previous COVID-19 compared with controls.^27,28^ In our cohort, we only analyzed standard morphofunctional parameters (IVSd, LViD, EDV, LAV, LVEF, TAPSE, PASP) so our results are limited to the parameters analyzed.

### Limitations

This study presents some limitations:

(1) It is retrospective and observational
(2) Relatively small sample size
(3) It is not possible to estimate the time from SARS-CoV2 asymptomatic or paucisymptomatic infection to the time of the clinical and echocardiographic examination
(4) Some studies suggested that even in patients with normal LVEF and TAPSE subclinical echocardiographic signs of myocardial dysfunction after COVID-19 might be detected, that is, studies using 2D speckle tracking. In our cohort, we only analyzed standard morphofunctional parameters (IVSd, LViD, EDV, LAV, LVEF, TAPSE, PASP) so our conclusions are limited to the parameters analyzed.

## Conclusion

The COVID-19 pandemic has had an important direct and indirect impact on health also because of the general reduction in health services with possible consequences on treatment outcomes and long waiting lists. As the emergency phase is passing, and more and more knowledge is being created about the COVID-19 disease and its possible consequences, it is, therefore, now mandatory to limit inappropriate diagnostic performances to reconvert the limited health resources towards interventions of proven efficacy.

Among 133 pediatric patients with a history of COVID-19 asymptomatic/mild illness, follow-up echocardiographic examinations showed no significant abnormalities in morphofunctional parameters. We can conclude that children who have experienced an asymptomatic or mild SARS-CoV-2 infection should not be systematically screened for cardiac involvement with second-level techniques, including TTE, in the absence of suggestive clinical symptoms or signs or other risk conditions such as congenital heart diseases, comorbidities, or risk factors.

### Conflicts of interest

There are no conflicts of interest.
